# Long‐term impact of obesity: Unraveling adipose epigenetic memory

**DOI:** 10.1002/ctm2.70254

**Published:** 2025-03-04

**Authors:** Laura C. Hinte, Daniel Castellano‐Castillo, Ferdinand von Meyenn

**Affiliations:** ^1^ Department of Health Sciences and Technology Laboratory of Nutrition and Metabolic Epigenetics, Institute of Food, Nutrition and Health Zurich Switzerland; ^2^ Department of Medical Oncology Virgen de la Victoria University Hospital Málaga Biomedical Research Institute (IBIMA)‐CIMES‐UMA Málaga Spain

**Keywords:** Adipose Tissue, Cellular Memory, Epigenetics, Obesity, Weight Loss

## 1 INTRODUCTION AND CHALLENGES IN OBESITY MANAGEMENT

Obesity and its associated comorbidities represent a global health burden, with over 1 billion individuals currently living with obesity.^1^ Thus, a primary objective in addressing the obesity pandemic, alongside preventative measures related to lifestyle and environmental factors, is achieving body weight reduction and long‐term body weight stability. Therapeutic approaches range from dietary and lifestyle interventions to bariatric surgery and pharmacological treatments, such as incretin mimetics. However, short‐term dietary and lifestyle interventions frequently do not achieve sustained weight loss and culminate in weight regain—the so‐called yo‒yo effect. Even incretin mimetics (e.g., semaglutide and tirzepatide), which have demonstrated impressive efficacy in body weight reduction and improvement of metabolic health, fail to maintain weight stability following withdrawal in humans. The human body's tendency to defend increased body weight makes weight loss and maintenance challenging, potentially due to a phenomenon akin to a ‘metabolic memory’, reminiscent of the ‘legacy effect’ observed in diabetes. This metabolic memory may be mediated by a plethora of mechanisms such as structural physiological changes, alterations in synaptic plasticity,^2^ gut microbiome,^3^ immune system,^4^, ^5^ vasculature,^6^ genetic contributions, and notably, epigenome, all of which warrant further investigation.

## 2 EPIGENETIC MEMORY OF OBESITY AND ADIPOSE TISSUE

Epigenetic mechanisms are essential for cellular development, differentiation and identity maintenance, and they are increasingly implicated as crucial contributors to a memory of obesity.^7^ Yet, it remained unresolved whether individual cells retain a metabolic memory of obesity and whether epigenetic mechanisms are involved in this. To address this question, we focused on the adipose tissue. Adipose tissue, a metabolically active organ, is heavily influenced by obesity and weight loss. As adipocytes are long‐lived (approximately 10 years)^8^ and non‐dividing cells, they serve as an ideal model for studying epigenetic memory. Additionally, adipose tissue is easily accessible for human biopsies, enabling longitudinal studies not feasible for most other organs.

We collected human omental and abdominal subcutaneous adipose tissue biopsies from three European study centres in Germany and Sweden. Specifically, we obtained samples from individuals living with obesity but without diabetes, both before and 2 years after bariatric surgery (RYGB or sleeve gastrectomy), as well as from healthy normal‐weight donors from the same centres. Only individuals who had lost at least 25% of their body mass index within 2 years were included. Using these biopsies, we performed single nucleus RNA sequencing (snRNA‐seq) to obtain transcriptional data for each cell type in the adipose tissue. A comparative analysis revealed that genes deregulated during obesity compared to controls often remained deregulated even after substantial weight loss. Adipocytes, precursor cells and vascular cells displayed the most persistent transcriptional changes, indicating that adipose tissue retains cell‐type‐specific transcriptional changes despite appreciable weight loss and a return to a normal‐weight state.^9^

Given the inherent limitations of human studies, such as genetic and environmental variability, we next extended our analysis to mice. Obesity was induced in mice through a high‐fat diet (HFD) and reversed by switching them back to a standard chow diet. Unlike dieting humans, mice rapidly lost weight, reaching body weights comparable to age‐matched controls within 4‒8 weeks. They mostly exhibited normal metabolic function, including liver fat clearance, normalised insulin and leptin levels, and restored energy expenditure. Despite these apparent phenotypic recoveries, snRNA‐seq analysis of their adipose tissue revealed that adipocytes, endothelial cells and precursors retained a transcriptional memory of obesity, consistent with our human data. This finding confirmed that this transcriptional memory of obesity is not exclusive to humans.

Further analysis of epigenetic marks (H3K4me3, H3K27me3, H3K4me1, H3K27ac) and chromatin accessibility of already existent adipocytes prior to HFD exposure demonstrated widespread chromatin remodelling in adipocytes during obesity. Many obesity‐induced changes persisted post‐weight loss. For example, promoters that are active in mice that never experienced obesity remained silenced, and the enhancer landscape was remodelled, confirming that adipocytes retain an epigenetic memory of obesity. This persistent state could predispose formerly obese individuals to faster weight regain and accelerated adipose tissue expansion when exposed to obesogenic conditions again.

**FIGURE 1:**
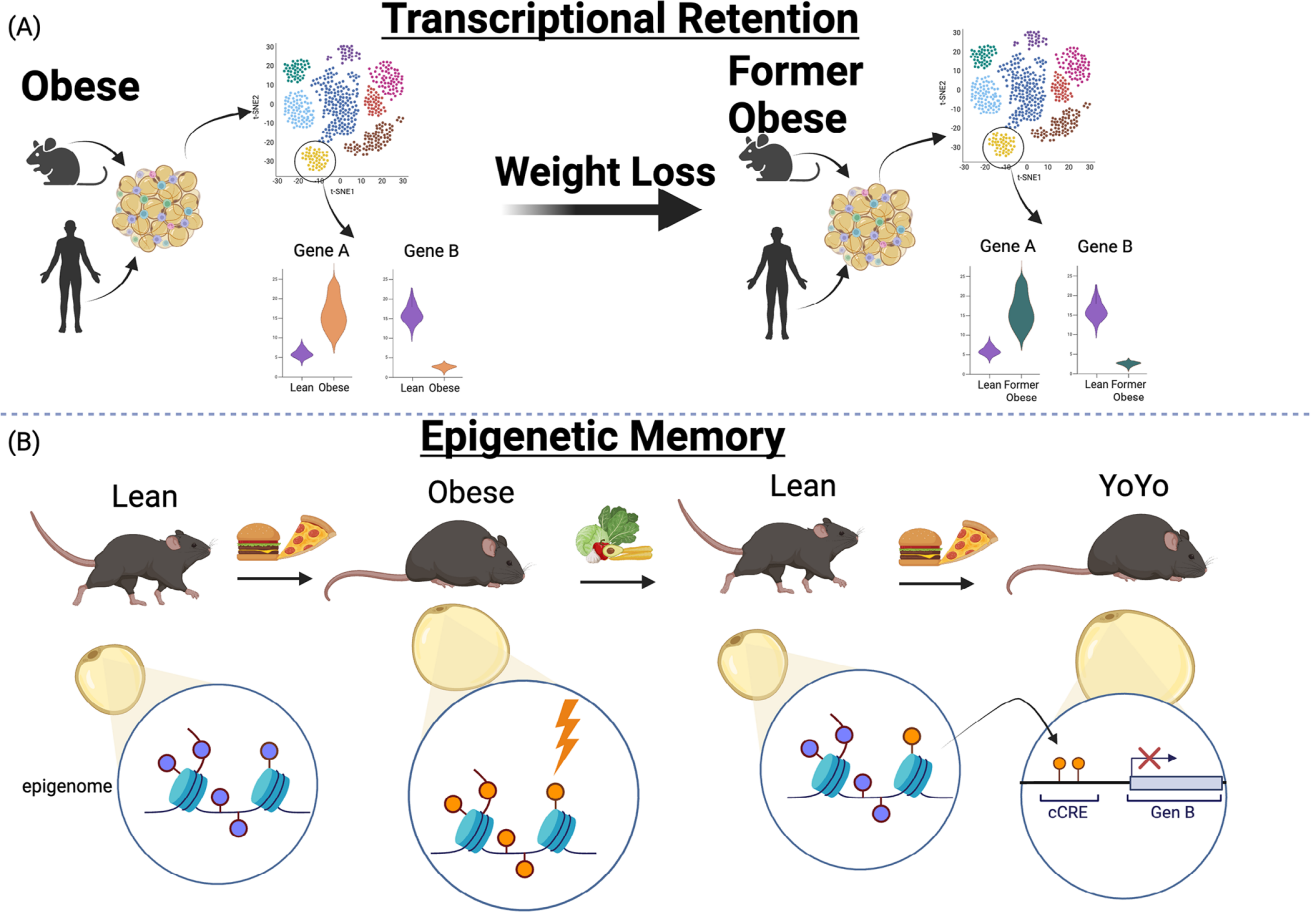
Schematic illustration of obesogenic transcriptional retention and epigenetic memory of cells in the adipose tissue (created with Biorender.com). (A) Human and mouse adipose tissue from was subjected to single nucleus RNA sequencing (snRNA‐seq). Comparative gene expression analysis prior to and post‐weight loss revealed retention of obesity‐induced transcriptional changes in cells of the murine and human adipose tissue. (B) The epigenome of murine adipocytes is remodelled during obesity and only partially returns to a lean state post‐weight loss. Adipocytes retain an epigenetic memory which can be linked to transcriptional regulation after a rechallenge with obesogenic stimuli.

When we challenged these memory‐retaining adipocytes with palmitate and glucose ex vivo, they exhibited a faster response compared to controls. Similarly, mice regained weight more rapidly when reintroduced to a HFD, with their adipose tissue expanding at an accelerated rate. Notably, the epigenetic memory could be used to predict differential gene expression in adipocytes after the rechallenge. These findings suggest that epigenetic memory predisposes formerly obese individuals to regain weight more easily, highlighting the long‐term consequences of obesity on adipose tissue function. This is one of the first studies to demonstrate that specific cell types can retain an epigenetic memory of a prior metabolic state (Figure 1).

## 3 CLINICAL IMPLICATIONS AND FUTURE DIRECTIONS

Clinically, these findings challenge stigmatising narratives around obesity by providing a biological basis for weight regain, independent of behavioural factors. They also highlight the necessity for sustained interventions—pharmacological and lifestyle‐based—to counteract the resistance imposed by metabolic memory. Moreover, leveraging epigenetic diagnostics could enable stratification of individuals based on their propensity for weight regain, opening the way for personalised therapies, including targeted epigenomic editing to reprogram obesity‐associated cellular memory.

The discovery of epigenetic memory in adipose tissue prompts exploration of similar phenomena in other cell types, such as hypothalamic neurons involved in appetite regulation. These cells, being long‐lived and non‐dividing, may also retain a memory through persistent chromatin changes. For example, studies in cocaine‐addicted rats have shown lasting alterations in chromatin accessibility—a proxy for epigenetic changes—within neurons,^10^ suggesting parallels in chronic conditions rooted in cellular memory. Investigating these mechanisms could open novel therapeutic avenues for obesity and related diseases, emphasising the broader relevance of epigenetic memory in chronic disease management.

## AUTHOR CONTRIBUTIONS

Laura C. Hinte, Daniel Castellano‐Castillo and Ferdinand von Meyenn conceptualised and wrote the commentary.

## CONFLICT OF INTEREST STATEMENT

The authors declare they have no conflicts of interest.

## ETHICS STATEMENT

Not applicable.
